# Rab32 interacts with SNX6 and affects retromer-dependent Golgi trafficking

**DOI:** 10.1371/journal.pone.0208889

**Published:** 2019-01-14

**Authors:** Dieter Waschbüsch, Nicole Hübel, Edith Ossendorf, Evy Lobbestael, Veerle Baekelandt, Andrew J. Lindsay, Mary W. McCaffrey, Amir R. Khan, Angelika Barnekow

**Affiliations:** 1 Department of Experimental Tumorbiology, Westfalische Wilhelms University Muenster, Muenster, Germany; 2 School of Biochemistry and Immunology, Trinity Biomedical Sciences Institute, Trinity College Dublin, Dublin, Ireland; 3 Laboratory for Neurobiology and Gene Therapy, KU Leuven, Leuven, Belgium; 4 School of Biochemistry and Cell Biology, Biosciences Institute, University College Cork, Cork, Ireland; Institut Curie, FRANCE

## Abstract

The Rab family of small GTPases regulate various aspects of cellular dynamics in eukaryotic cells. Membrane trafficking has emerged as central to the functions of leucine-rich repeat kinase 2 (LRRK2), which is associated with inherited and sporadic forms of Parkinson’s disease (PD). Rabs act as both regulators of the catalytic activity and targets for serine/threonine phosphorylation by LRRK2. Rab32, Rab38 and Rab29 have been shown to regulate LRRK2 sub-cellular localization through direct interactions. Recently, Rab29 was shown to escort LRRK2 to the Golgi apparatus and activate the phosphorylation of Rab8 and Rab10. Rab32 is linked to multiple cellular functions including endosomal trafficking, mitochondrial dynamics, and melanosome biogenesis. A missense mutation in Rab32 has also recently been linked to PD. Here, we demonstrate that Rab32 directly interacts with sorting nexin 6 (SNX6). SNX6 is a transient subunit of the retromer, an endosome-Golgi retrieval complex whose Vps35 subunit is strongly associated with PD. We could further show that localization of cation-independent mannose-6-phosphate receptors, which are recycled to the *trans*-Golgi network (TGN) by the retromer, was affected by both Rab32 and SNX6. These data imply that Rab32 is linked to SNX6/retromer trafficking at the Golgi, and also suggests a possible connection between the retromer and Rab32 in the trafficking and biological functions of LRRK2.

## Introduction

Rab small GTPases comprise the largest member of the Ras superfamily with over 60 proteins in mammalian cells [1]. Rab oscillate between GTP and GDP-bound forms to regulate various aspects of membrane trafficking including vesicle transport, cytoskeletal and organelle dynamics. In their active (GTP) state, local conformation transitions in their switch 1 and switch 2 regions enable recruitment of effector proteins that subsequently regulate cellular dynamics. The small GTPase Rab32 regulates endosomal pathways associated with biogenesis of lysosome-related organelles (LRO) such as melanosomes or in microbial host defense [2–4]. Rab32 regulates these cellular pathways through interacting proteins that include the AP-1 and AP-3 adaptor protein complexes, and the retromer-interacting protein VARP[5]. However, Rab32 is also found in cells lacking specialized organelles such as melanosomes, and there is evidence for a role in endosomal transport and mitochondrial dynamics [6]. Rab32 and Rab38, which is closely related in sequence and function, also control mitochondrial fission by interacting with the mitochondrial fission factor Drp1 [7, 8].

It has emerged that the Rab GTPases play a central role in regulating the functions of Parkinson’s disease (PD)-associated leucine-rich repeat kinase 2 (LRRK2). Parkinson’s disease is a disorder of the central nervous system that involves progressive degeneration of motor functions and deposition of fibrils termed Lewy bodies in the cytoplasm. Late- and early-onset forms of the disease are linked to multiple genes, with the most prevalent being *LRRK2*. The 2,527-residue protein product contains a kinase domain towards the C-terminus that phosphorylates Ser/Thr residues. Preceding the kinase domain are two tandem domains: the Ras of complex (ROC) domain that binds GTP/GDP and is distantly related to a Rab GTPase, and the C-teminal of ROC (COR) domain. These tandem domains regulate the kinase activity and they are found in proteins with diverse cellular functions, thus they are grouped into the eponymously named ROCO protein family [9].

Rab32, Rab38 and the closely related Rab29 have all recently been associated with interactions to LRRK2 and subsequent effects on its sub-cellular localization [10, 11]. Rab29 recruits LRRK2 to the Golgi and activates the kinase, leading to enhanced phosphorylation of Rab8 and Rab10 in their switch 2 region [12]. Rab32 is not a target for the kinase but it interacts with LRRK2 and regulates its sub-cellular localization [11]. Thus, Rabs control the catalytic functions of LRRK2 and they are also substrates for the enzyme, mediating downstream effects that may be important for an understanding of the pathology observed in PD. Mutations in the Vps35 subunit of the retromer complex (Vps26.Vps35/Vps29), which regulates endosomal sorting, are also linked to PD. Recently, the pathogenic D620N mutation of Vps35 has been shown to enhance the phosphorylation of Rab8, Rab10 and Rab12 by LRRK2[13]. The link between the retromer and LRRK2 activity, in the context of Parkinson’s disease, remains unknown.

Here we demonstrate a physical interaction between Rab32 and SNX6, which is a transient component of the retromer [14, 15]. SNX6 is composed of an N-terminal lipid/protein binding pox homology (PX) domain followed by a BAR domain that induces or stabilizes curved membrane bilayers. We demonstrate that Rab32 and Rab38 physically interact with SNX6 through its PX domain. Although there was only limited co-localization of the interacting proteins, we can demonstrate the influence of Rab32 on retromer-mediated transport processes, such as the transport of Shiga toxin subunit B (STxB) to the Golgi apparatus, as well as the transport of cation-independent mannose-6-phosphate receptors (CI-MPR).

## Material and methods

### Plasmids and cloning

The pAS2-Rab32^WT^ construct has previously been described [11]. Constitutively active and inactive constructs were made by PCR using the following primers: forward: AGAATTCCATATGGCGGGCGGAGGAGCC; reverse: TACCTAGGTCAGCAA-CACTGGGATTTGTTC using pDsRed-Monomer constructs of the respective mutants as template. The plasmids for GST-pulldown were derived from these pAS2 plasmids by sub-cloning. The GST-Rab1A^WT^ plasmid was also made by sub-cloning from a formerly described construct [16]. The Rab38 constructs also have been previously described [11]. pACT-SNX6ΔC in the initial Yeast two-hybrid screen was found in a human lung library (Clonetech, Mountain View, CA, USA). It covers the first 195 residues of human SNX6 with the mutation K191N. All other SNX6ΔC constructs used in this study were derived from this construct by sub-cloning. pGADT7-SNX6 (full length SNX6) was constructed by sub-cloning from two plasmids, pACT-SNX6ΔC and a pECFP-SNX6 which was a kind gift from José Javier Fuster (Fuster et al., 2010). This was necessary, because different variants of SNX6 exist, and the ECFP construct was different at the N-terminus of the protein (12 residues shorter). The SNX6 construct used in this study resembles the transcript variant 2 (NM_152233) and does not contain the K191N mutation. All other full length SNX6 constructs were derived from this plasmid by sub-cloning. All other SNX full length constructs were amplified by PCR using the human lung library as template. The primers were designed to have NdeI and BamHI sites for cloning into the pACT2 vector. The coding sequences were from the following database entries: SNX1—NM_003099; SNX2—NM_003100; SNX5—NM_152227; SNX6—NM_152233; SNX32—NM_152760. Constructs pET15b-Rab38^WT^ (1–181) and pET28b-SNX6 (PX domain, residues 1–193) were ordered from Genscript (Piscataway, NJ, USA). The sequences were optimized for expression in *E*. *coli*. All constructs in this study made by PCR were confirmed by sequencing.

### Cell culture and transfection

Culture of IHKE-1 cells has been described before [11]. Cells were grown in DMEM/Ham’s F12 supplemented with 1% FBS, 15 mM HEPES (pH 7.2), 44 mM NaHCO3, 1 mM sodiumpyruvate, 4.5 mM L-glutamine, 36 ng/ml hydrocortisol, 10 ng/ml EGF, 5 μg/ml insulin, 5 μg/ml transferrin and 5 ng/ml sodium selenite. Transfections with either Turbofect (Thermo Scientific/Pierce, Rockford, USA) or K2 (Biontex Laboratories, Munich, Germany) were carried out according to the manufacturers protocol. SH-SY5Y cells stably expressing GFP-LRRK2 were described before [17]. The cells were cultured at 37°C, 5% CO_2_ in DMEM supplemented with 10% FBS and 1% non-essential amino acids.

### Antibodies

Mouse anti SNX6 (D-5) was obtained from Santa Cruz Biotechnology and used at a 1:1000 dilution in Western blotting experiments and 1:100 in secondary immunofluorescence. Other antibodies for immunofluorescence were: Mouse anti Rab6A (5B10, produced in house) at a dilution of 1:50; Mouse anti cation-independent mannose-6-phosphate receptor (2C2) was a generous gift from Regina Pohlman, UKM, Münster, Germany and used at a dilution of 1:100; The ‘Prestige’ Rabbit anti Rab32 antibody by Atlas antibodies was from Sigma, St. Lois, MO, USA and used at 1:50 dilution. The Rabbit anti sorting nexin 1 antibody (Thermo Fisher, Rockford, IL, USA) was used at a dilution of 1:100. Secondary antibodies against either mouse or rabbit were coupled to Oyster594, Oyster 647 or Oyster405 (Luminartis GmbH, Muenster, Germany), cy2, cy3, Alexa350, Alexa488, Alexa594 or Alexa647 (Jackson Immunoresearch, Newmarket, UK or Cell Signaling Technology, Leiden, Netherlands). The following antibodies were used in Western blotting: Rabbit anti LRRK2 (c41-2) MJFF2 (abcam, Cambridge, UK) diluted 1:2000; The mouse anti GFP antibody (JL-8, Clontech, Mountain View, USA) was used at a dilution of 1:4000. Rabbit anti Rab32 SAB4200086 (Sigma, St. Lois, MO, USA) was used at a dilution of 1:1500, the Mouse anti Rab38 A-8 (Santa Cruz Biotechnology, Dallas, Texas, USA) at 1:1000. HRP-coupled anti-rabbit and anti-mouse secondary antibodies were obtained from Cell Signaling Technology (Danvers, MA, USA) and used diluted 1:1000.

### Western blotting

Western Blotting procedure was described before [11]. In brief, an SDS-Page was run and the transfer to a PVDF membrane was done by a semidry system applying a constant current. The transfer was controlled by Ponceau S staining followed by blocking in 5% skimmed milk powder in PBS-T (0.1% Tween 20). LRRK2 antibodies were incubated overnight at 4°C, other antibodies were incubated for 1 hour at RT on a shaker. Secondary antibodies were coupled to horseradish peroxidase and the signal was detected by X-ray film for appropriate times depending on the signal strength.

### GST-pulldowns, direct pulldowns and immunoprecipitation

GST-pulldown procedure was carried out as previously described [11]. In this paper, we used lysates from human cell lines as prey lysates as indicated in the text. Co-immunoprecipitation was carried out using the GFP-Trap kit (Chromotek, Planegg-Martinsried, Germany). Transfectected IHKE-1 cells were harvested in a modified GFP-Trap buffer (10mM Tris pH7.5, 150mM NaCl, 0.5mM EGTA, 0.5% NP40, 200μM sodium orthovanadate, complete EDTA free). The lysates were cleared by centrifugation. The co-immunoprecipitation procedure was carried out according to manufacturer instructions.

For the direct pulldown experiments 6his-SNX6 (PX domain) and 6his-Rab38^WT^ (1–181) were expressed in *E*. *coli* BL-21(DE3) by induction with 0.5mM IPTG. Cells were grown in 1l 2YT medium at 37°C until the OD at 600nm was 0.7 ± 0.1. Then temperature was switched to 18°C and recombinant protein expression was induced by addition of 0.5 mM IPTG. The cells were further incubated at 18°C over night. The cells were harvested by centrifugation and proteins were extracted by sonication of the resuspended pellets in 10mM Tris pH8, 300mM NaCl, 10 mM Imidazole, 5mM MgCl2 and 10 mM β-Mercaptoethanol. Resuspended cells were lysed by sonication and clarified by centrifugation at 25,000 x *g*. The supernatant was loaded to a Ni-NTA agarose resin (Thermo Scientific, Rockfort, IL, USA) in a gravity-flow column column for immobilized metal affinity chromatography (IMAC). After washing the column with extraction buffer (additional rigourous wash step with 40 mM Imidazole) the His-tagged proteins were eluted with buffer containing 200mM Imidazole containing buffer. The SNX6-PX domain construct was cleaved over night using 20 IU Thrombin in dialysis against gel filtration buffer (10 mM Tris pH7.5, 100mM NaCl, 5mM MgCL_2_, 1mM DTT). Remaining uncleaved protein was removed by applying the solution to a reverse IMAC step. Both bait and prey proteins were further purified by gel filtration on an superdex 200 16/60 column (GE, Uppsala, Sweden). The cleaved and uncleaved purified proteins were concentrated in 10 kDa MWCO concentrator tubes (Merck Millipore (Amicon), Carrigtwohill, Ireland). Concentrations were determined using a NanoDrop spectrometer (Thermo Scientific, Wilmington, DE, USA). 6his-Rab38^WT^ (1–181) was incubated with a 5x molar excess of GppNHp (GTP analogue) or GDP (Jena Bioscience, Jena, Germany) and EDTA for 15 min at room temperature to enable the exchange of the initially bound nucleotide. The exchange reaction was quenched by addition of excess MgCl_2_. Excess nucleotide was removed by gel filtration (superdex 200 10/300, GE Healthcare Uppsala, Sweden). 1 mg of GppNHp or GDP bound 6his-Rab38^WT^ (1–181) was mixed with 1mg of SNX6 (PX domain) in extraction buffer (control without the bait protein). 25 μl of Ni-NTA agarose resin was added followed by rotating at 4°C for 15 minutes. The Ni-NTA agarose was harvested by gentle centrifugation (1000 xg, 2 minutes, 4°C). The resulting pellet was washed 3 x with extraction buffer followed by elution of the proteins in 20 μl of 4 x loading dye (95°C, 5 minutes). 1 μl of the elute was analyzed by SDS-PAGE.

### Immunofluorescence and microscopy

Secondary immunofluorescence procedure was carried out as previously described [11]. In short, cells were grown on NaHCO_3_ treated glass cover slips. Whether the cells were transfected transiently or not, they were washed in ice cold PBS 24–48 hours after seeding or transfection and subsequently fixed in 4% PFA in PBS for 30 minutes. Cells were permeabilized for 5 minutes in 0.2% Triton X100 solution and then blocked with 10% goat serum in PBS containing 0.2% gelatine. PBS-gelatine was also used to dilute and incubate primary and secondary antibodies, which were incubated for 1 hour or 20 minutes, respectively. Cells were mounted in MOWIOL supplemented with DABCO and DAPI and analyzed with a Leitz Diaplan microscope equipped with PL-Fluotar lenses (50x, 1.0 N.A. and 100x, 1.32 N.A.) and filters optimized for GFP (excitation: 450–490 nm, emission: 515–560 nm), TexasRed (excitation: 540–580 nm, emission: 607–682 nm) and DAPI fluorescence. Images were taken with an Olympus XM10 camera as 16 bit multipage .tif files. Images from all channels were captured with a 2 second exposure and automatic adjustment of brightness and contrast on screen by the Olympus Cell^F software. Structured illumination microscopy (SIM) was carried out on a CellObserver (Carl Zeiss, Jena, Germany) equipped with ELYRA S.1 system using a 63 x Plan Apochromat 1.4 N.A. objective. The processed images were saved as 16bit multipage .tif files. The 16 bit images from both SIM and epifluorescence microscope were processed in ImageJ [18]. Brightness and contrast were adjusted for best visibility. We also used a Zeiss LSM5 for fluorescence microscopy. Here, we adjusted detector gain and laser power to obtain 8bit multipage .tiff files. Processing and assembly of the final images were also done in ImageJ.

### Yeast two-hybrid

The human lung Matchmaker cDNA Library (Clontech, Mountain View, CA, USA) was a kind gift from Stefan Ludwigs Lab, UKM, Muenster, Germany. Co-transformation of the reporter yeast strain Y190 (Clontech, Heidelberg, Germany) was described previously [19]. Co-transformation of the reporter strain Gold (Clontech, Heidelberg, Germany) was done similar to the Y190 strain according to the manufacturer’s instructions.

### STxB transport assay

In this assay the Shiga toxin subunit B coupled to the fluorophore Cy3 (STxB-cy3) was used to monitor retromer mediated transport from early endosomes to the trans-Golgi-network bypassing late endosomes [14]. The Cy3 conjugated STxB was a kind gift from L. Johannes, Institute Curie, Paris, France). The experiment was performed in HeLa cells (ATCC CCL-2.2) which were kept in DMEM with 10% FKS. 100,000 cells were seeded to 35 mm cell culture dishes with 12 mm glass cover slips. After 24 hours, cells were transfected with plasmids encoding GFP-Rab6A’(Q72L), GFP-Rab6A’(T27N), GFP-Rab32(Q85L) or GFP-Rab32(T39N) and incubated for another 48 hours. STxB-cy3 was diluted to a final concentration of 1 ng/μl in DMEM containing 15 mM HEPES. Cells were washed 2x with ice cold PBS. The STxB-cy3 DMEM solution was placed in 60mm cell culture dishes in 20 μl drops so the cover slips with the HeLa cells could be placed face down in them. The dishes were incubated at 4°C for 30 minutes to allow the STxB-cy3 to bind the cell surface. After washing twice with ice cold PBS, internalization to early endosomes was triggered by incubation at 19.5°C for 60 minutes. Incubation at that temperature prevents further transport of the internalized STxB-cy3. To start retromer mediated transport, cells were transferred back to 37°C for 60 minutes. Then, cells were washed twice with warm PBS and fixed in 4% PFA in PBS for 20 minutes at 37°C. Immunofluorescence staining of endogenous Rab6 as a Golgi-apparatus marker was performed according to our immunofluorescence protocol. The secondary antibody for Rab6 detection was coupled to Oyster405, a blue fluorescent dye. Images were taken with the Leitz Diaplan microscope and the 50 x 1.0 N.A. Objective.

We quantified the overlap of internalized STxB-cy3 with the Rab6-Golgi stain compared to the total STxB-cy3 signal to get a measure for its transport. Therefore, we used the ImageJ software[19]. First, the images were blurred by a 2-pixel median filter followed by background subtraction using a rolling ball radius of 20 pixels. We determined the borders of a transfected cell by using the polygon tool to get rid of STxB-cy3 signal from surrounding cells. The STxB-cy3 channel and the Golgi channel were now binarized to black or white. In the resulting overlay image, the size of yellow areas (= co-localization; A_co-localization_) were determined using the wand tool. The respective value for total STxB-cy3 area coverage (A_STxB-cy3_) was determined from the binarized STxB-cy3 channel image. The overlap was calculated by the following formula: Overlap in % = A_co-localization_/A_STxB-cy3_)*100.

#### CI-MPR trafficking assay

The localization analysis of the cation-independent mannose-6-phosphate receptor (CI-MPR) has previously been described [20]. In brief, IHKE-1 cells were seeded on glass cover slips and 24 hours later were transfected with the indicated plasmids. Additionally, stable IHKE cell lines expressing GFP (control), GFP-Rab32^WT^ or GFP-Rab32 Q85L were used. After a further 24 hours, cells were fixed and immunofluorescently stained for CI-MPR. Images of randomly selected fields were recorded with the Leitz Diaplan microscope with the PL Fluotar 50x 1.0 N.A. The CI-MPR channel was separated after marking the transfected cells. These cells were visually divided into two classes: compact CI-MPR localization (at the TGN), and dispersed CI-MPR pattern throughout the cytoplasm. All cells expressing the same recombinant proteins from the individual experiments were pooled for statistical analysis. The significance was determined by a Fisher’s Exact Test, a variant of the chi-squared test.

## Results and discussion

**Fig 1:**
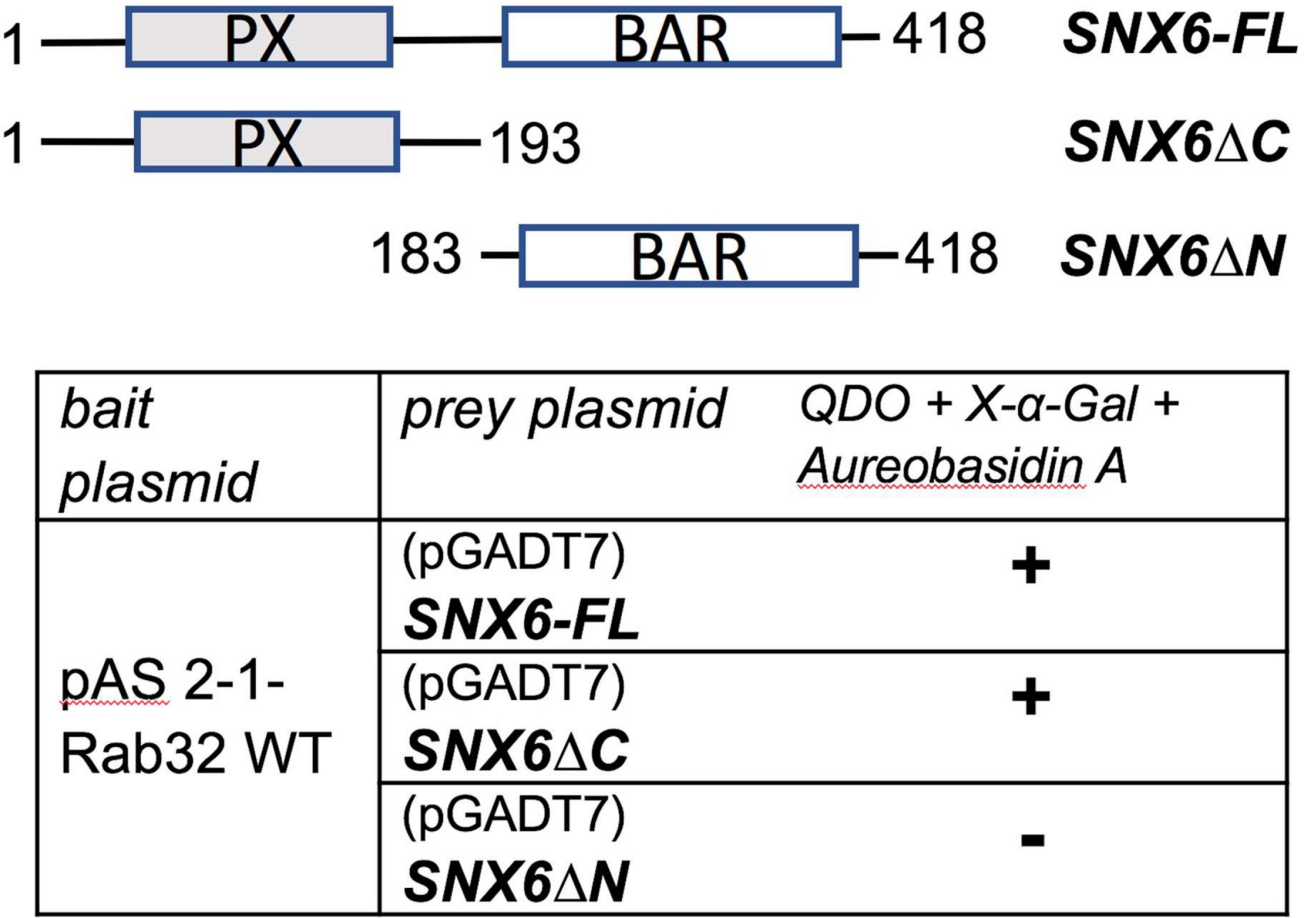
Rab32 interacts with the PX domain of SNX6. Rab32-interacting domains were identified by constructing different plasmids containing either full length SNX6 or the BAR (SNX6ΔN) or PX (SNX6ΔC) domains only. Co-transformations were peformed in the yeast strain Gold with the indicated plasmids. Colony growth on QDO plates and blue color indicates that the proteins interact (+), n≥3 independent experiments.

**Fig 2:**
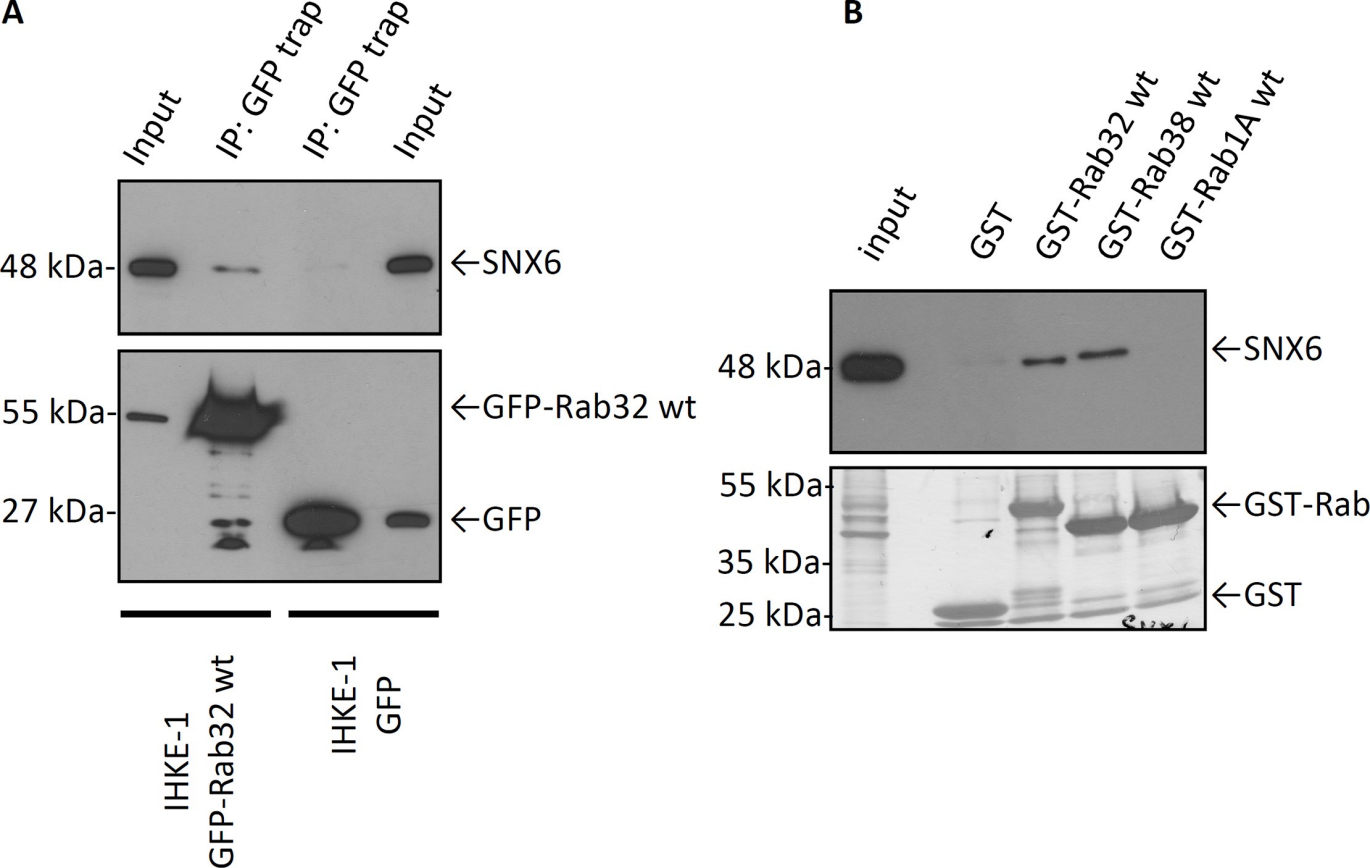
Rab32, Rab38 and SNX6 binding in co-immunoprecipitation and GST-*pulldown* experiments. (A) Lysates from IHKE-1 cells stably expressing GFP-Rab32 were used for co-immunoprecipitation of endogenous SNX6 with the GFP-Trap kit. IHKE-1 cells transiently transfected with GFP alone were used as control. n = 2 independent experiments. (B) 5 μg GST-Rab32 wt, GST-Rab38 wt, GST-Rab1A wt or GST as control were loaded to glutathione agarose beads followed by incubation with IHKE-1 lysate overnight. Samples were analyzed by SDS-PAGE and subsequent Western blot analysis against SNX6. n = 3 independent experiments.

We previously reported that LRRK2 is a novel interacting partner of the small GTPase Rab32 using a yeast two hybrid screen [11]. Using the same approach, we show that Rab32 interacts with SNX6, a transient member of the retromer complex (**Table 1**). In our screen we identified a PX-domain containing fragment of SNX6, SNX6ΔC, as the region that interacts with Rab32 (**Fig 1**). To confirm the interactions, we performed co-immunoprecipitation experiments using the GFP-Trap kit. As demonstrated in **Fig 2A** we were able to co-precipitate endogenous SNX6 from IHKE-1 cells transfected with GFP-Rab32 wt, but not from cells expressing GFP only. We were able to pull down endogenous SNX6 from IHKE-1 cells with GST-Rab32 and GST-Rab38 constructs but not with GST alone or GST-Rab1A as GTPase control (**Fig 2B**).

**Table 1:** Yeast two-hybrid analyses of SNX6 binding to Rab GTPases.

bait plasmid	prey plasmid	QDO + X-α-Gal + Aureobasidin A
pAS 2-1-Rab32 Q85L	pGADT7-SNX6	**+**
pAS 2-1-Rab38 Q69L		**+**
pAS 2-1-Rab6B Q72L		-
pAS 2-1-Rab6A Q72L		-
pAS 2-1-Rab6A‘ Q72L		-
pAS 2-1-Rab27B Q78L		-
pAS 2-1-Rab11A		-
pAS 2-1-Rab1B Q67L		-
pAS 2-1-Rab1A Q70L		-
pAS 2-1-Rab2 Q65L		-
pAS 2-1-Rab5A		-
pAS 2-1-Rab4A		-
pAS 2-1-Rab7ΔC		-
pAS 2-1-Rab9ΔC		-

In order to test if SNX6 specifically interacts with Rab32, the indicated plasmids were co-transformed with the yeast strain Gold. Colony growth on QDO plates and blue color indicates that the proteins interact (+). Only Rab32 and the closely related Rab38 tested positive for interactions with SNX6. n≥3 independent experiments.

The retromer complex is a heteropentameric complex that consists of a VPS trimer and a SNX dimer [15]. The latter is made of SNX1 or SNX2 in complex with either SNX5, SNX6 or SNX32, which are all members of the SNX-BAR subfamily. To test, whether the interactions with Rab32 and Rab38 were specific for SNX6 we performed yeast two hybrid protein-protein interaction assays with other members of the SNX family. Only SNX6 can bind to Rab32/38—all other SNXs tested fail to activate reporter genes in the yeast cells (**Table 2**).

**Table 2:** Sorting nexin binding specificity for Rab32 and Rab38.

bait plasmid	prey plasmid	ß-Gal
pAS 2-1-Rab32 wt	pGADT7-SNX1	-
	pGADT7-SNX2	-
	pGADT7-SNX5	-
	pGADT7-SNX6	**+**
	pGADT7-SNX32	-
pAS 2-1-Rab38 wt	pGADT7-SNX1	-
	pGADT7-SNX2	-
	pGADT7-SNX5	-
	pGADT7-SNX6	**+**
	pGADT7-SNX32	-

Rab32 and Rab38 were co-transformed with the yeast strain Gold using the indicated plasmids. Colony growth on -AT plates and blue color in β-Gal filter lift assay indicated that the proteins interact (+). Interactions are specific for SNX6, n≥2 independent experiments.

We also investigated, whether the interaction of Rab32 and Rab38 with SNX6 is GTP- dependent. In all cases, the wildtype and constitutively active mutants were able to bind SNX6 in co-transformation experiments and GST-pulldown experiments (**S1 Table and S1 Fig**). In contrast, the GDP-locked mutants of Rab32 and Rab38 did not activate reporter genes in the yeast two hybrid assays, but were able to bind SNX6 in GST-pulldown experiments. Therefore the issue of GTP/GDP dependence of binding remains unclear, but it is possible that the GTP conformation of Rab32/38 binds with greater affinity to SNX6.

### Co-localization analyses of SNX6 and Rab32

**Fig 3:**
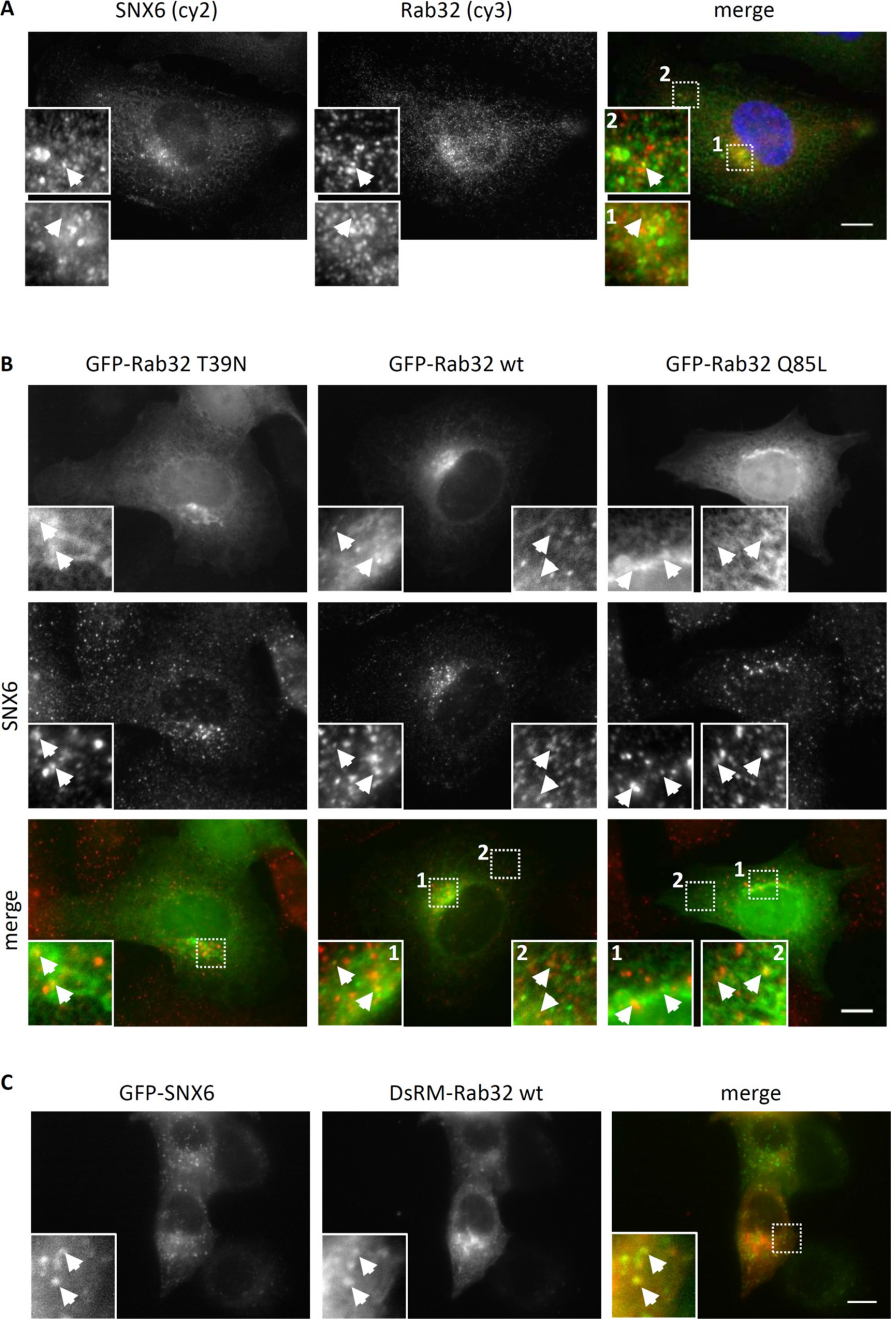
Co-localization analysis of SNX6 with endogenous, constitutively active, inactive and wild-type Rab32. (A) IHKE-1 cells were cultured on glass cover slips for 24 hours, fixed and subsequently stained with antibodies against Rab32 (cy3, red channel) and SNX6 (cy2, green channel). Blue: DAPI staining of the nucleus. (B) IHKE-1 cells were either transiently transfected with plasmids encoding GFP-Rab32 T39N or stably expressing GFP Rab32^WT^ or GFP-Rab32 Q85L, fixed and subsequently stained with an antibody against SNX6. GFP = green channel; SNX6 (cy3) = red channel (C) HeLa cells cultured on glass cover slips were transfected with constructs encoding GFP-SNX6 or DsRed-Monomer-Rab32 wt. After 24h cells were fixed and analyzed by flourescece microscopy. GFP = green; DsRed-Monomer = red. Arrows indicate co-localization, scale bar = 10μm.

We next set out to determine whether the Rab32 and SNX6 co-localize in the cell. We analyzed the sub-cellular localization of the endogenous proteins by immunofluorescence. To insure the specificity of the antibodies we tested them as described in **S1 Document and S2–S4 Figs**. Both proteins localize to small vesicular structures, but only a relatively small number of co-localizing punctae were observed (**Fig 3A**). The insets show, that this pattern of co-localization is found directly at a structure that we describe as pericentrosomal recycling endosomes (inset 1). We also observed co-localization of Rab32 and SNX6 in the cell periphery (inset 2).

Because of the relatively low level of co-localization, we looked for changes in number, size/area and frequency of the co-localization patterns by analyzing co-localization of overexpressed GFP-Rab32 constructs with endogenous SNX6. It is apparent that co-localization of endogenous SNX6 with GFP-Rab32 WT is similar to endogenous Rab32 (**Fig 3B**). We further observe that the inactive mutant, GFP-Rab32 T39N, although very diffuse shows some co-localizing vesicles with SNX6. We also find co-localizing punctae with GFP-Rab32 Q85L in the perinuclear area (inset 1) of the cells and in the periphery (inset 2). Overexpressing both DsRed-Monomer-Rab32^WT^ and GFP-SNX6 revealed a higher degree of co-localization which coincides with the increased abundance of the interacting proteins (**Fig 3C**). We further wanted to know whether the SNX6 bound to Rab32 is might be involved in retromer functions. Therefore, we performed immunofluorescence labelling of GFP-Rab32^WT^ and GFP-Rab32 Q85L with both SNX6 and SNX1. It is evident from **S5 Fig** that a least a proportion of Rab32 co-localizining to SNX6 also co-localizes with SNX1. From these data, we suspect that Rab32 may be involved in retromer-mediated trafficking.

#### Rab32 and SNX6 influence Shiga toxin trafficking

We tested whether Rab32 was able to influence SNX6 transport by analyzing a typical retromer cargo such as cation independent mannose-6-phospate receptors (CI-MPR) or the Shiga toxin subunit B (STxB) [21, 22]. STxB is a subunit of the bacterial toxin from *Shigella dysenteriae* which is endocytosed to early endosomes [14, 23]. From there direct retromer mediated transport to the Golgi apparatus happens as by knockdown of retromer components like Vps26 or SNX1 was shown [21]. It was furthermore demonstrated, that the constitutively inactive mutant of Rab6A’ was able to reduce/inhibit the retromer mediated transport of STxB and therefore was used as control in our experiment [24].

**Fig 4:**
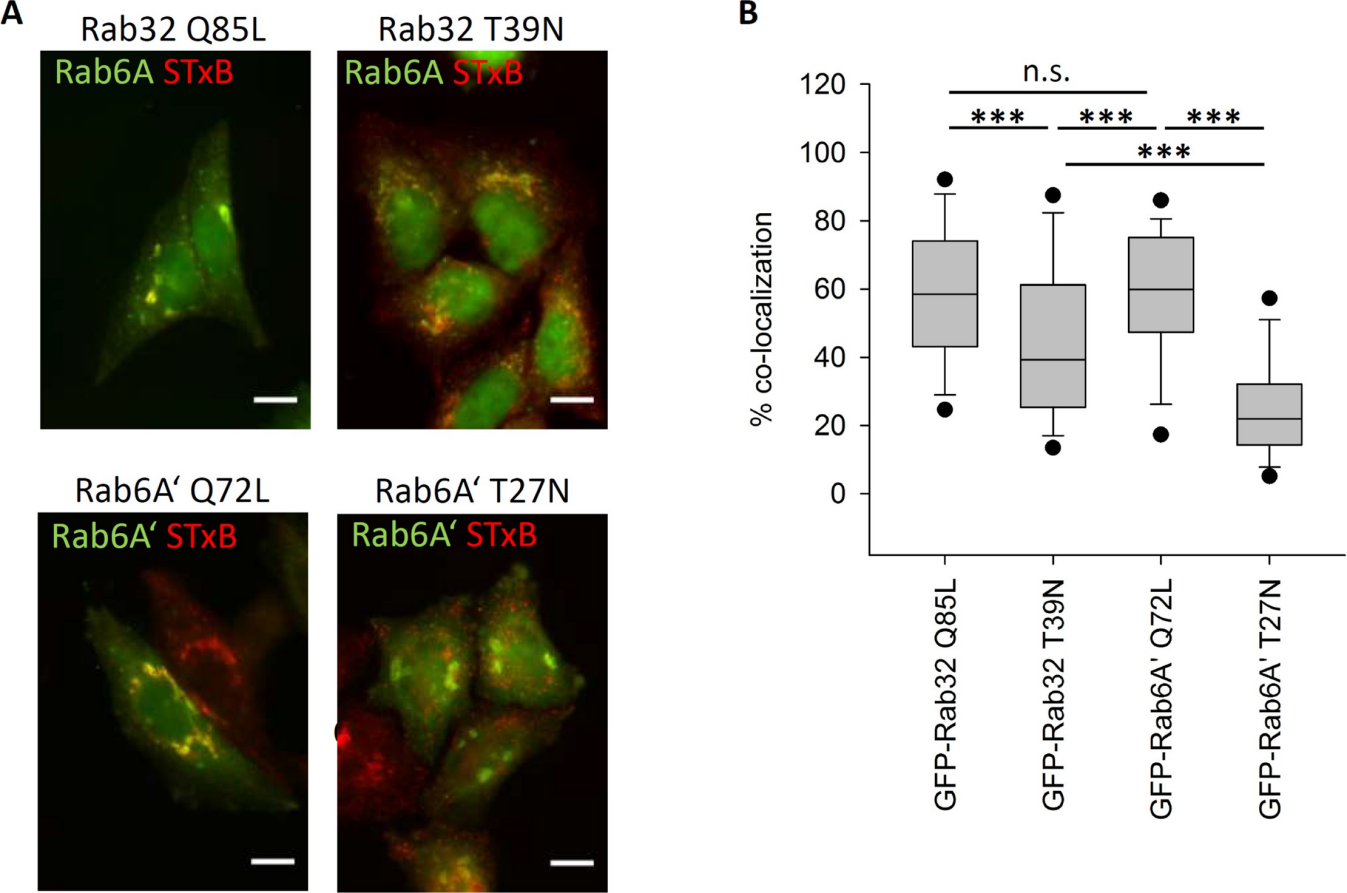
Rab32 influence on retromer mediated transport of Shiga toxin B. (A) HeLa cells transfected with either GFP-Rab32 Q85L, GFP-Rab32 T39N (upper row), GFP-Rab6A’ Q72L or GFP-Rab6A’ T27N (lower row). 24 hours later cells were incubated with STxB-cy3 and pulse-chased for 60 minutes, followed by fixation in 4% PFA. Rab32-expressing cells were additionally stained by immunofluorescent labeling of Rab6A. (blue) to have a comparable Golgi marker to the GFP-Rab6A’ transfected cells. The Rab6A/A’ channels were depicted in green, STxB-cy3 in red. STxB co-localizing with the Golgi apparatus appears in yellow. GFP-Rab32 constructs are not visible. Scalebar = 10 μm (B) Quantification of STxB transport rates. Endogenous Rab6A or GFP-Rab6A’ was used to determine the localization of the Golgi apparatus. Transport rate is given as % co-localization of STxB with the Golgi apparatus. A lower % co-localization indicates a reduced rate of STxB transport. Rab32 Q85L: n = 127 cells, Rab32 T39N: n = 119 cells, Rab6A’ Q72L: n = 132 cells; Rab6A’ T27N: n = 131 cells.

The vast majority of STxB-cy3 co-localizes with the Golgi-apparatus in the cells transfected with the constitutively active mutants of Rab32 and Rab6A’ (**Fig 4A**). The median co-localization was 58% for GFP-Rab32 Q85L and 59% for GFP-Rab6A’ Q72L (**Fig 4B**). In contrast, in cells overexpressing GFP-Rab32 T39N and GFP-Rab6A’ T27N, a proportion of STxB-cy3 remains in the cell periphery where there is no apparent co-localization with the Golgi-apparatus (**Fig 4A**). The control, GFP-Rab6A’ T27N expressing cells, display only 22% overlap. In cells expressing GFP-Rab32 T39N, the co-localization with the Golgi apparatus is also reduced significantly compared to the constitutively active Rabs—only 44.3% of the STxB-cy3 in median co-localizes with the Rab6A signal (**Fig 4B**). It can be concluded that overexpression of an inactive mutant of Rab32 causes a reduction in retromer-mediated STxB transport similar to Rab6A’.

#### Rab32 and SNX6 influence CI-MPR trafficking

**Fig 5:**
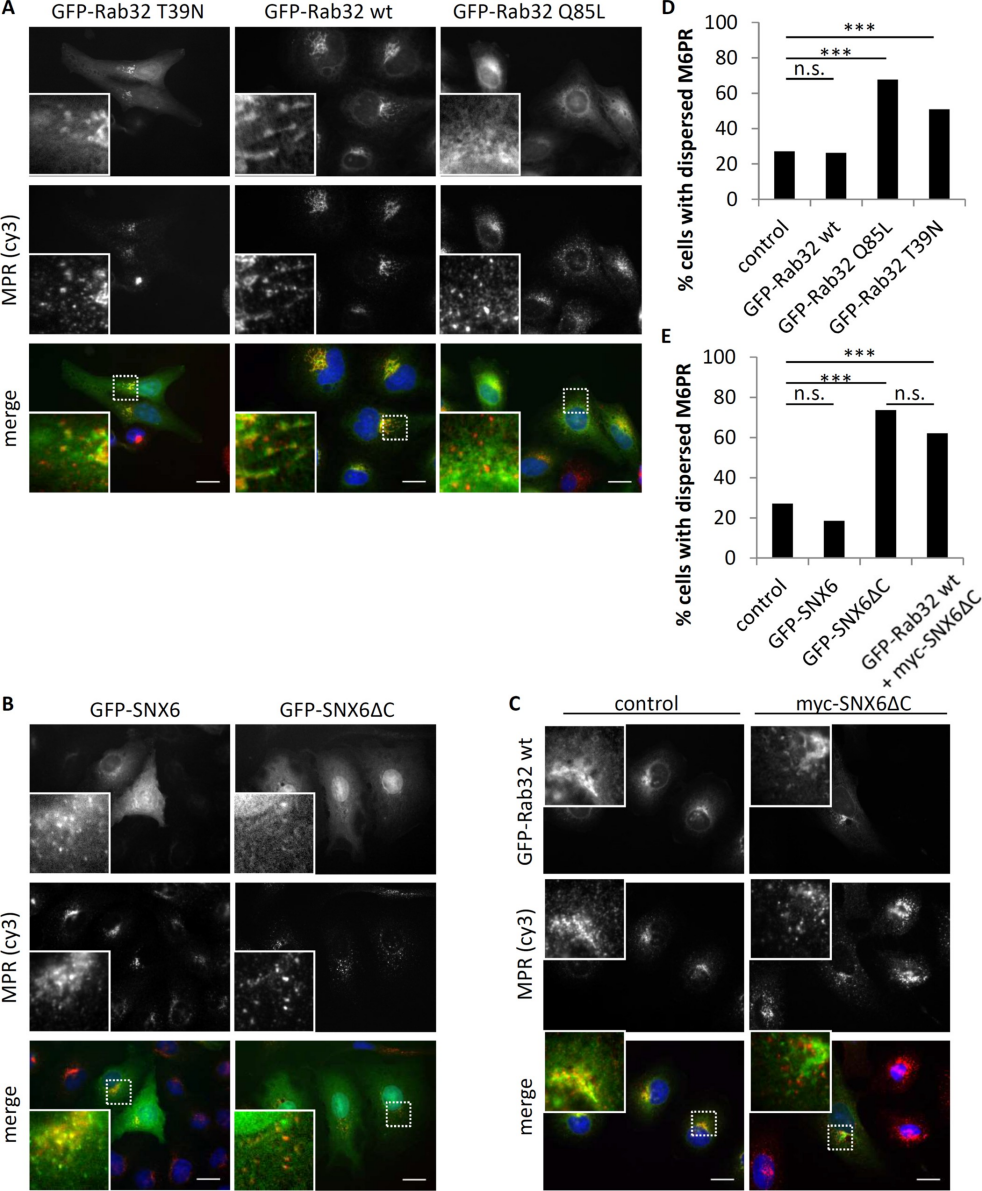
Rab32 and SNX6 influence on cation independent mannose-6 phosphate receptor (CI-MPR) trafficking in IHKE-1 cells. (A) IHKE-1 cells transfected with either GFP-Rab32 T39N, GFP-Rab32^WT^ or GFP-Rab32 Q85L. After 24 hours cells were fixed in 4% PFA and subsequently stained for CI-MPR. (B) IHKE-1 cells transfected with either GFP-SNX6 or GFP-SNX6ΔC. After 24 hours, cells were fixed in 4% PFA and subsequently stained for CI-MPR. (C) IHKE-1 cells transfected with either GFP-Rab32^WT^ or GFP-Rab32^WT^ and myc-SNX6ΔC. After 24 hours cells were fixed in 4% PFA and subsequently stained for CI-MPR. Successful double transfection was assesed by immunofluorence against myc-tag (not shown) demonstrating >95% co-transfected cells. (D) Quantification of CI-MPR distribution in the cell for different Rab32 constructs. MPR-signal was either categorized as dispersed phenotype or not disperesed. n for control, GFP-Rab32^WT^, -Q85L or -T39N were 910, 210, 275 and 110 cells from ≥3 independent experiments. (E) Quantification of CI-MPR distribution in the cell for different SNX6 constructs MPR-signal was either categorized as dispersed phenotype or not dispersed. n for control, GFP-SNX6, GFP-SNX6ΔC or Rab32 wt+SNX6ΔC were 910, 54, 57 and 124 cells from ≥2 independent experiments. Significance was determined by Fisher’s Exact Test; p<0.005 = ***, n.s. = not significant; Scalebar = 10 μm.

To further investigate the role of Rab32 in retromer function, we analyzed the localization and distribution of cation independent mannose-6-phosphate receptors (CI-MPR) in IHKE-1 cells upon overexpression of different Rab32 and SNX6 constructs. In GFP-Rab32^WT^ expressing IHKE-1 cells, co-localization with CI-MPR was observed in a perinuclear structure that resembles the Golgi-apparatus or TGN (**Fig 5A and S6 Fig**). This observation was also seen in cells with GFP-Rab32 T39N expression. In contrast, there was some but less co-localization of CI-MPR with GFP-Rab32 Q85L at the Golgi membrane. Furthermore, the pattern of CI-MPR seems to be more dispersed in the cytoplasm of the cells compared to cells expressing the other Rab32 constructs. However, the integrity of the Golgi apparatus itself does not seem to be affected (**S6 Fig**). In cells expressing GFP-SNX6 there is also co-localization with CI-MPR (**Fig 5B**), which is in good agreement with earlier studies[25]. The GFP-SNX6ΔC construct has a different appearance compared to full length SNX6: it looks more diffuse and a proportion of it is found in the nucleus. Furthermore, the CI-MPR appear dispersed, as also observed for GFP-Rab32 Q85L expressing cells. When we co-express GFP-Rab32 with myc-SNX6ΔC we obtain a reduced co-localization of CI-MPR and GFP-Rab32 WT indicating a role for SNX6 in CI-MPR retrieval (**Fig 5C**).

We also analyzed the distribution of the receptor by classifying its appearance as ‘dispersed’ or ‘not dispersed’ as described in earlier reports [20, 26]. As seen in **Fig 5D**, only 27% of untransfected control or 26% GFP-Rab32^WT^ expressing IHKE-1 cells exhibit a dispersed CI-MPR pattern. The constitutively active GFP-Rab32 Q85L has a much higher number of cells with dispersed CI-MPR (68%). The inactive GFP-Rab32 T39N expressing cells also show an increase in cells with dispersed CI-MPR (51%). When expressing GFP-SNX6 the number of cells with dispersed CI-MPR is fairly low (19%), but upon expression of GFP-SNX6ΔC, 73% of all cells show dispersed CI-MPR comparable to GFP-Rab32(Q85L) expressing cells (**Fig 5E**). The co-expression of GFP-Rab32^WT^ did not result in a rescue of the myc-SNX6ΔC induced dispersed CI-MPR phenotype (62% dispersed CI-MPR). Therefore, we concluded that Rab32 affects CI-MPR trafficking in a SNX6-dependent manner by either reducing CI-MPR retrieval or increased transport towards endosomes.

#### Triple co-localization of GFP-LRRK2, Rab32 and SNX6

**Fig 6:**
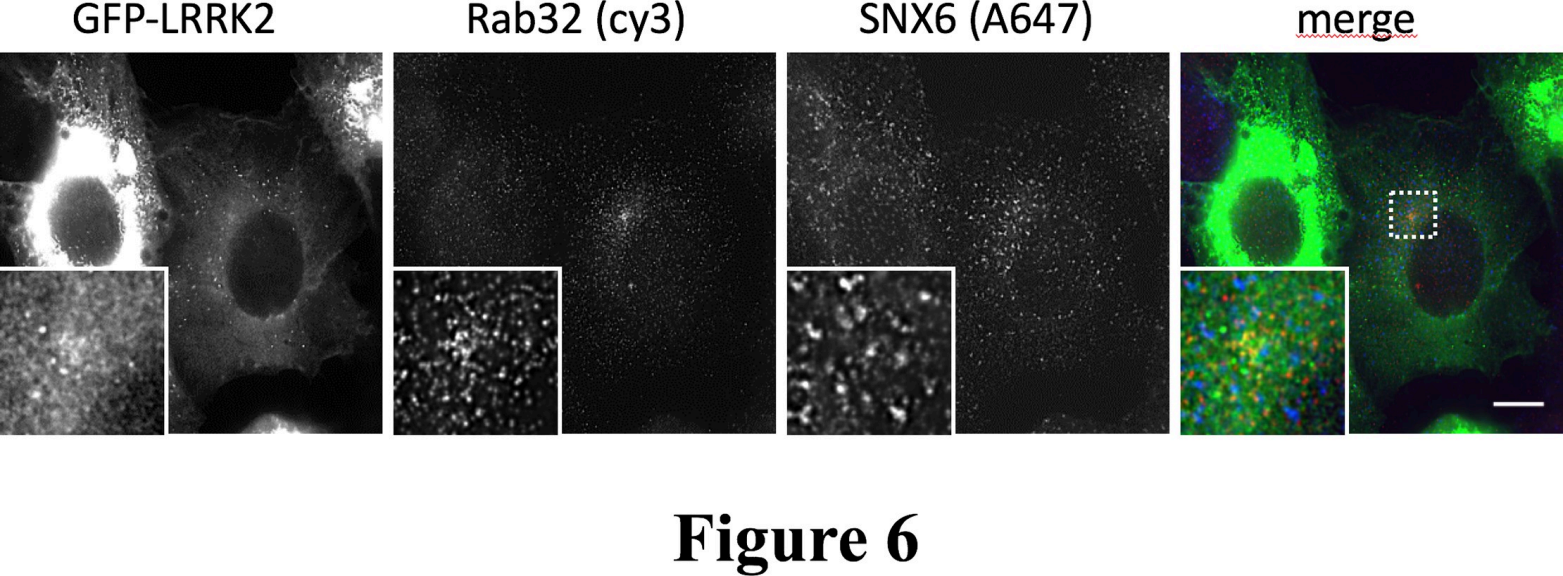
Co-localization analysis of GFP-LRRK2 with endogenous Rab32 and SNX6. SH-SY-5Y stably expressing GFP-LRRK2 cells were cultured on glass cover slips for 48 hours. Then, cells were stained with antibodies against Rab32 and SNX6. Secondary antibodies were coupled to cy3 or Alexa 647. Cells were analyzed wit a Zeiss SIM microscope. n = 2 independent experiments. Colors in the merge image: GFP = green, cy3 = red, Alexa 647 = blue color; Scale bar = 10μm.

The Rab32 related protein Rab29 has been shown to mediate retrograde trafficking of CI-MPR, maintain the integrity of the TGN, and modify retromer-dependent sorting in a PD-associated cell model[27, 28]. This transport also involves LRRK2 and the retromer, demonstrated by overexpressing the PD mutants LRRK2-G2019S and Vps35-D620N or knockout of the retromer component Vps35. LRRK2 and SNX6 are both Rab32 interacting proteins, therefore we performed triple co-localization experiments. SH-SY5Y cells stably expressing GFP-LRRK2 were fixed and subjected to immunofluorescence staining against Rab32 and SNX6. Because a triple co-localization could neither be confirmed nor rejected by conventional laser scanning microscopy (**S7 Fig**), we used the structured illumination microscopy (SIM) super resolution technique. The images display little or no triple co-localization (**Fig 6**), therefore it remains unclear whether Rab32 can simultaneously engage SNX6 and LRRK2.

## Conclusions

Here we demonstrate that Rab32 and Rab38 directly bind to SNX6 through its PX domain. Wild-type and GTP-locked variants of Rab32/38 reveal interactions using both pulldowns and yeast two-hybrid analyses. However, the GDP-locked variant of Rab32/38 fails to interact with SNX6 in the yeast two-hydrid assays. Since the wild-type forms of Rab GTPases are likely to have mixtures of GTP/GDP during the assay, the results imply a stronger interaction between SNX6 and the GTP conformations of Rab32/38. The interactions with SNX6 are specific–no other sorting nexins show binding to Rab32/38. Although there is little co-localization, transport assays involving SNX6/retromer dependent STxB-cy3 cargo and the analysis of CI-MPR localization reveal that both SNX6 and Rab32 are involved in retrograde trafficking of retromer dependent cargo.

Our previous work revealed a direct interaction between Rab32/38 and the PD-associated LRRK2 protein[11]. Rab-mediated trafficking pathways are becoming central to an understanding of the biological functions of LRRK2. Rab29 recruits LRRK2 to the Golgi and enhances LRRK2 phosphorylation of Rab8 and Rab10 in their switch 2 α-helices [12]. The Phosphorylation of Rab8 enables recognition by Rab-interacting lyososmal protein-like 2 (RILPL2), leading to increased ciliogenesis[29]. The binding sites for Rab32/38 and Rab29 remain unclear, although a model has been suggested that places Rab32/38 at the N-terminal armadillo domain (1–552) [11, 12]. The triple co-localization experiments failed to detect appreciable amounts of ternary complexes of SNX6:Rab32:LRRK2. However, there is a precedent for Rab engagement of two interacting proteins simultaneously—Rab11 binds to both Rabin8 and FIP3 to regulate ciliogenesis[30]. The molecular details of Rab32 interactions with SNX6 and LRRK2 require further characterization.

LRRK2 is also involved in retromer-mediated trafficking of CI-MPR, and overexpression of PD-related LRRK2 mutants interfere with this process[27]. A widespread Vps35 mutation (D620N) is strongly associated with early-onset PD [10, 20, 31], and recently this mutation has been observed to activate the kinase activity of LRRK2 through an unknown intermediate[13]. A recent report suggests that SNX6 may also mediate retromer-independent sorting of CI-MPR[32], therefore the links between Rab32 and the retromer require further investigation. Intriguingly, the retromer has become associated with neuroprotection, and perturbation of this novel role may be linked to LRRK2 mutations and subsequent pathologies seen in PD[33]. However, the novel interactions between SNX6, Rab32/38 and LRRK2 are interesting given the recent identification of a Rab32 missense mutation linked to PD[34, 35]. The effect of the mutation on interactions with Rab32-interacting proteins may shed light on the links between Rab32 and PD. Despite the absence of clear connections, the ensemble of evidence is intriguing, suggesting that Rab32/38, sorting nexins and the retromer regulate signaling pathways upstream of LRRK2 kinase activation. Future studies will require the detailed mapping of these interactions and how they influence LRRK2 phosphorylation of substrate Rab GTPases.

## Supporting information

S1 Document File containing information regarding the validation of Rab32 antibody used in the experiments. (PDF)

S1 Fig Rab32, Rab38 and SNX6 binding in GST-*pulldown* experiments. (A) 5 μg GST-Rab32 Q85L, -T39N or GST as control were incubated with IHKE-1 cell lysates in the presence of GST-Trap beads. Samples were analyzed by SDS-PAGE and subsequent Western blot analysis against SNX6 and LRRK2. n ≤ 3 independent experiments. (B) Western Blot corresponding to **Fig 1B**: 5 μg GST-Rab32 wt, -Q85L, -T39N or GST as control were loaded to glutathione agarose beads followed by incubation with IHKE-1 lysate overnight. Samples were analyzed by SDS-PAGE and subsequent Western blot analysis against SNX6. n ≥ 3 independent experiments. (C) Western Blot, 5 μg GST-Rab38 wt, -Q69L, -T23N or GST as control were loaded to glutathione agarose beads followed by incubation with IHKE-1 lysate overnight. Samples were analyzed in by SDS-PAGE and subsequent Western blot analysis against SNX6. n ≥ 3 independent experiments.
(TIFF)

S2 Fig Specificity of the Rabbit anti Rab32 antibody HPA025731. IHKE-1 cells stably expressing GFP-Rab32 wt were grown on glass cover slips for 24 hours followed by fixation and subsequently stained with an Rabbit anti Rab32 antibody (HPA025731). Despite being ‘stable’ some cells lost the expression of GFP-Rab32 wt–visible endogenous Rab32 was indicated by the arrow. Scale bar 10 μm.
(TIF)

S3 Fig Specificity of the Rabbit anti Rab32 antibody HPA025731. (A) SH-SY5Y cells were either grown normally or in the presence of 10 μM retinoic acid to induce neuronal differentiation. After lysis Western blots against Rab32 (Rabbit andt Rab32 SAB4200086), Rab38 and GAPDH as loading control were performed. (B) Quantification of the Western blots shown in (A); n = 3 independent experiments (C) Seconday immunofluorescence of SH-SY5Y cells stably expressing GFP-LRRK2 (not shown) either undiffentiated or differentiated by retinoic acid for 7 days. The images were taken at a 2 seconds exposure as 16 bit .tif files and the images were adjusted equally (black value was set to 900, white to 5700 of a total range of 0 to 65535). This allows a visual comparison of the signal strength. Scale bar = 10 μm (D) siRNA knockdown of Rab32. IHKE-1 cells were transfected with either control or siRNA against Rab32 for 3 days. Then lysates were prepared and and Western blots were done against GAPDH as loading control (lower panel) and Rab32 using the HPA025731 antibody (upper panel).
(TIF)

S4 Fig Specificity of the Mouse anti SNX6 antibody D-5. A549 (upper panel) or IHKE-1 cells were grown on glass cover slips before being fixed and stained for SNX6. In order to test the sepcificity of the antibody we added 0,35μg 6his-SNX6_1-193_-construct to the primary antibody solution for 5 minutes. The control was without this protein. Both samples were incubated with the same amount of secondary antibody. Samples containing blocking protein and the respective controls were analyzed on a LSM5 microscope with equal settings for laser power, pinhole and detector gain. Scale bar = 10 μm; n = 3 independent experiments.
(TIF)

S5 Fig Co-localization analysis of Rab32, SNX6 and SNX1. (A) IHKE 1 cells stably expressing either GFP-Rab32 wt (upper panel) or GFP-Rab32 Q85L (lower panel) were grown for 24 hours on glass cover slips. Then cells were fixed and stained for SNX1 and SNX6. Green channel: GFP; Red channel: Alexa 594 (SNX1); Blue channel: Alexa 647 (SNX6). Scale bar = 10μm (B) A549 cells were grown on glass cover slips for 24 hour followed by transfection with plasmids to express either DsRed-Monomer-Rab32 wt or DsRed-Monomer-Rab32 Q85L (depicted in red). After another 24 hours the cells were fixed and immunofluorescently labelled for SNX1 (Alexa488; green channel) and SNX6 (Alexa 647; blue channel). Scale bar = 10μm;
(TIF)

S6 Fig Golgi integrity in GFP-Rab32^WT^ and GF-Rab32 Q85L expressing cells. IHKE-1 cells stably expressing either GFP-Rab32^WT^ or GFP-Rab32 Q85L wer grown on glass cover slips, fixed and immunofluorescently labelled against Giantin (red channel) and M6PR (blue channel). Scalebar = 20μm.
(TIF)

S7 Fig Co-localization analysis of GFP-LRRK2 with endogenous Rab32 and SNX6. SH-SY-5Y stably expressing GFP-LRRK2 cells were cultured on glass cover slips for 48 hours. Then, cells were fixed and stained with antibodies against Rab32 and SNX6. Secondary antibodies were coupled to cy3 or Alexa 647. Cells were analyzed wit a Zeiss LSM5 microscope. n = 2 independent experiments. Colors in the merge image: GFP = green, cy3 = red, Alexa 647 = blue; Scale bar = 10μm.
(TIF)

S1 Table Nucleotide specificity of Rab32 binding SNX6. In order to test whether constitutively active (Q85L) or inactive (T39N) mutants interact with SNX6, we co-transformed the yeast strain Gold with the indicated plasmids. Colony growth on QDO plates and blue color indicates that the proteins interact (+), n≥3 independent experiments.
(DOCX)

## Abbreviations

SNX6: sorting nexin 6
ER: endoplasmic reticulum
LRRK2: leucine-rich repeat kinase 2
TGN: *trans*-Golgi network
LRO: lysosome-related organelle
Vps35: vacuolar protein sorting-associated protein 35
