# Supplementary material for: Rab32 interacts with SNX6 and affects retromer-dependent Golgi trafficking
Source: PLoS One. 2019 Jan 14;14(1):e0208889. doi: 10.1371/journal.pone.0208889 (PMC6331118; doi:10.1371/journal.pone.0208889)
Supplement: S1 Document — (PDF) [file pone.0208889.s001.pdf]

Antibody validation

Immunofluorescence images of cells stained with the Rab32 antibody were published before [1]. However, to guarantee the specificity of the Rab32 antibody additional experiments were conducted. We grew IHKE-1 cells stably expressing GFP-Rab32 wt on glass cover slips followed by fixation and secondary immunofluorescence staining for Rab32. Microscopic analysis revealed a good overlap of the GFP-signal and the one from the antibody staining indicating the antibody binds to Rab32 (**Figure S2**). Interestingly, cells that lost a visible GFP-Rab32 wt expression display a signal of endogenous Rab32 similar to the GFP-Rab32 signal. To further confirm the specificity of the antibody we grew SH-SY5Y cells stably expressing GFP-LRRK2 and either differentiated them for 7 days in retinoic acid or keep them undifferentiated. We found out that Rab32 is expressed at a higher level in undifferentiated cells compared to the neuronal differentiated. In contrast, Rab38 is more abundant in differentiated cells (**Figure S3A** and **3B**). To see if this can also be seen in immunofluorescence, differentiated and undifferentiated SH-SY5Y cells were fixed and stained with the Rabbit anti Rab32 antibody (HPA025731). Although both specimens show a similar pattern of staining (the arrows in **Figure 3C** indicate the accumulation of Rab32 at the pericentrosomal recycling endosome) the overall signal strength is lower in differentiated SH-SY5Y cells as observed in Western blots before. These results also exclude a potential cross reactivity with Rab38. To further confirm the specificity of the antibody we knocked down Rab32 in IHKE1 cells by using siRNA. Three days after transfecting the siRNA a clear reduction of the band at ~25 kDa was observed when using the Rab32 siRNA (**Figure 3D**). Therefore, we concluded the antibody is suitable for our experiments.
The specificity of the SNX6 antibody was tested by blocking the antibody ba adding an excess of recombinantly expressed SNX6 to the antibody solution in secondary immunofluorescence experiments. When adding this protein, there is no detectable signal observed in neither A549 or IHKE-1 cells (**Figure S4**). A further indication is that the signal obtained with the SNX6 antibody used in this study is specific is that there is significant co-localization with SNX1 as described in previous publications [2]; [3].

Additional methods

Preparation of recombinant SNX6. The preparation of 6his $SNX6_{1-193}$ follows the protocol described in material and methods for direct pulldowns. The protein was isolated by Ni-NTA agarose from bacterial lysates as described. The eluted protein was further purified by size exclusion chromatography using a superdex 200 16/60 column (GE, Uppsala, Sweden). The purified protein was concentrated in a 10 kDa MWCO concentrator tube (Merck Millipore (Amicon), Carrigtwohill, Ireland). Concentration of the protein was determined photometrically.

Knockdown of Rab32. siRNA was used to inhibit Rab32 expression. In our experiment we used MISSION esiRNA (Sigma Aldrich, St. Louis, MO, USA) which consists of a heterogenous mixture of siRNA to improve targeting. Lipofectamine 2000 was used to transfect IHKE-1 cells according to the manufacturers standard protocol. Three days after transfection, cells were harvested directly in 4 x SDS-PAGE loading dye and was subsequently analyzed by Western blot.

Western blotting. The Western blots for Rab32 knockdown experiment were carried out similar to the ones described in material and methods with the following differences: Proteins were blotted to a nitrocellulose membrane at constantly 12V. The blotting buffer was 50 mM Tris, 40 mM Glycine, 0.037% SDS and 10% Ethanol. Blocking as well as incubation with primary and secondary antibodies occurred in in 5% skimmed milk powder in TBS-T (0.1% Tween20). Washing between the antibody

incubations was with TBS-T. After the secondary antibody incubation, the membrane was washed twice with TBS-t followed by washing with TBS. The signals were detected using an Odyssay system (LI-COR, Cambridge, UK). The secondary antibodies used were either anti-Rabbit IRDye800 or anti-Mouse IRDye680 system (LI-COR, Cambridge, UK) used at a 1 to 10,000 dilution.

1. Ortiz-Sandoval CG, Hughes SC, Dacks JB, Simmen T. Interaction with the effector dynamin-related protein 1 (Drp1) is an ancient function of Rab32 subfamily proteins. Cell Logist. 2014;4: e986399. doi:10.4161/21592799.2014.986399

2. Wassmer T, Attar N, Bujny M V., Oakley J, Traer CJ, Cullen PJ. A loss-of-function screen reveals SNX5 and SNX6 as potential components of the mammalian retromer. J Cell Sci. 2006;120: 45–54. doi:10.1242/jcs.03302

3. Hong Z, Yang Y, Zhang C, Niu Y, Li K, Zhao X, et al. The retromer component SNX6 interacts with dynactin p150 Glued and mediates endosome-to-TGN transport. Cell Res. 2009;19: 1334–1349. doi:10.1038/cr.2009.130
