# Supplementary figures and images for: Rab32 interacts with SNX6 and affects retromer-dependent Golgi trafficking
Source: PLoS One. 2019 Jan 14;14(1):e0208889. doi: 10.1371/journal.pone.0208889 (PMC6331118; doi:10.1371/journal.pone.0208889)

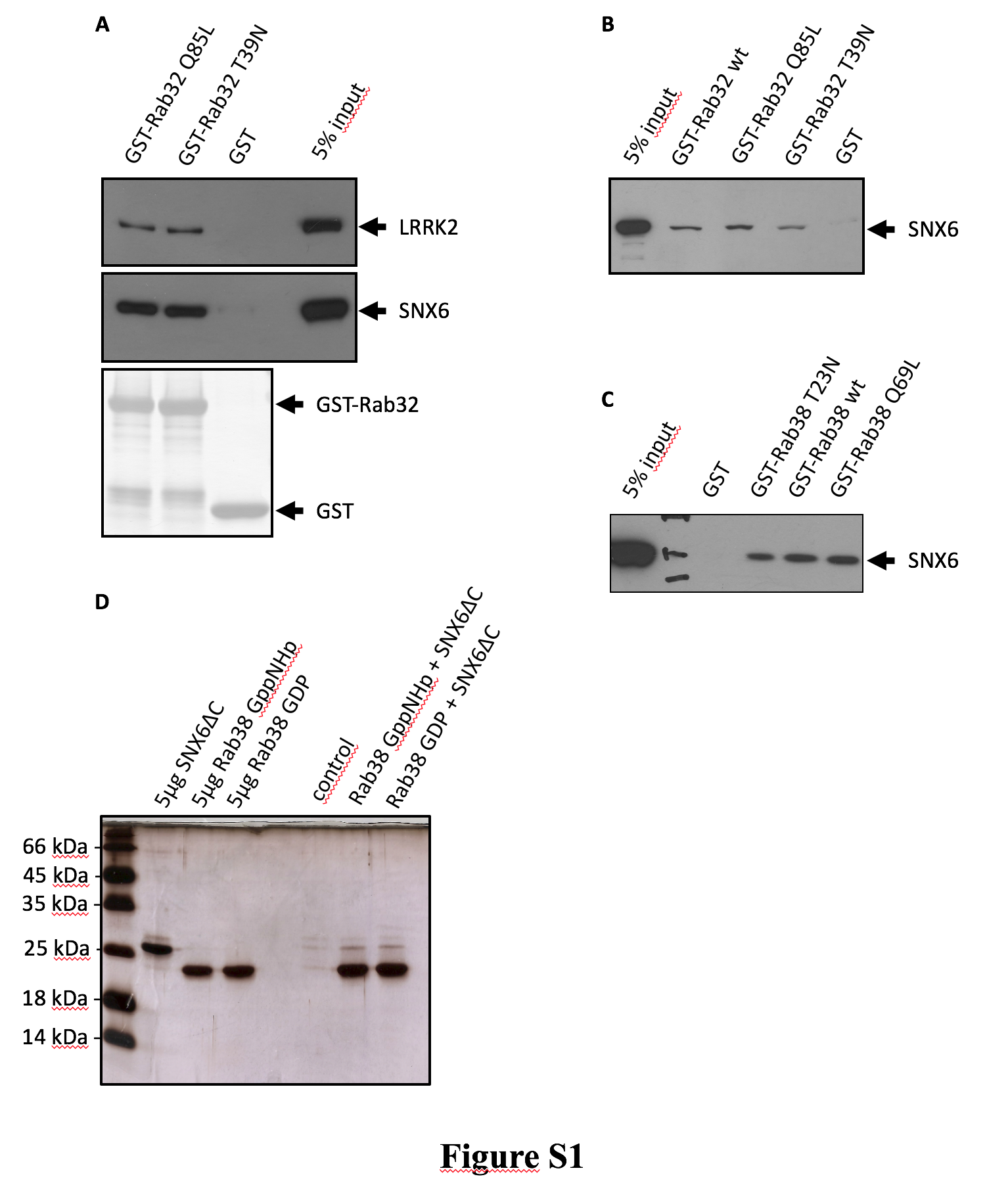

Supplement: S1 Fig — (A) 5 μg GST-Rab32 Q85L, -T39N or GST as control were incubated with IHKE-1 cell lysates in the presence of GST-Trap beads. Samples were analyzed by SDS-PAGE and subsequent Western blot analysis against SNX6 and LRRK2. n ≤ 3 independent experiments. (B) Western Blot corresponding to Fig 1B: 5 μg GST-Rab32 wt, -Q85L, -T39N or GST as control were loaded to glutathione agarose beads followed by incubation with IHKE-1 lysate overnight. Samples were analyzed by SDS-PAGE and subsequent Western blot analysis against SNX6. n ≥ 3 independent experiments. (C) Western Blot, 5 μg GST-Rab38 wt, -Q69L, -T23N or GST as control were loaded to glutathione agarose beads followed by incubation with IHKE-1 lysate overnight. Samples were analyzed in by SDS-PAGE and subsequent Western blot analysis against SNX6. n ≥ 3 independent experiments. (TIFF) [file pone.0208889.s002.tiff]

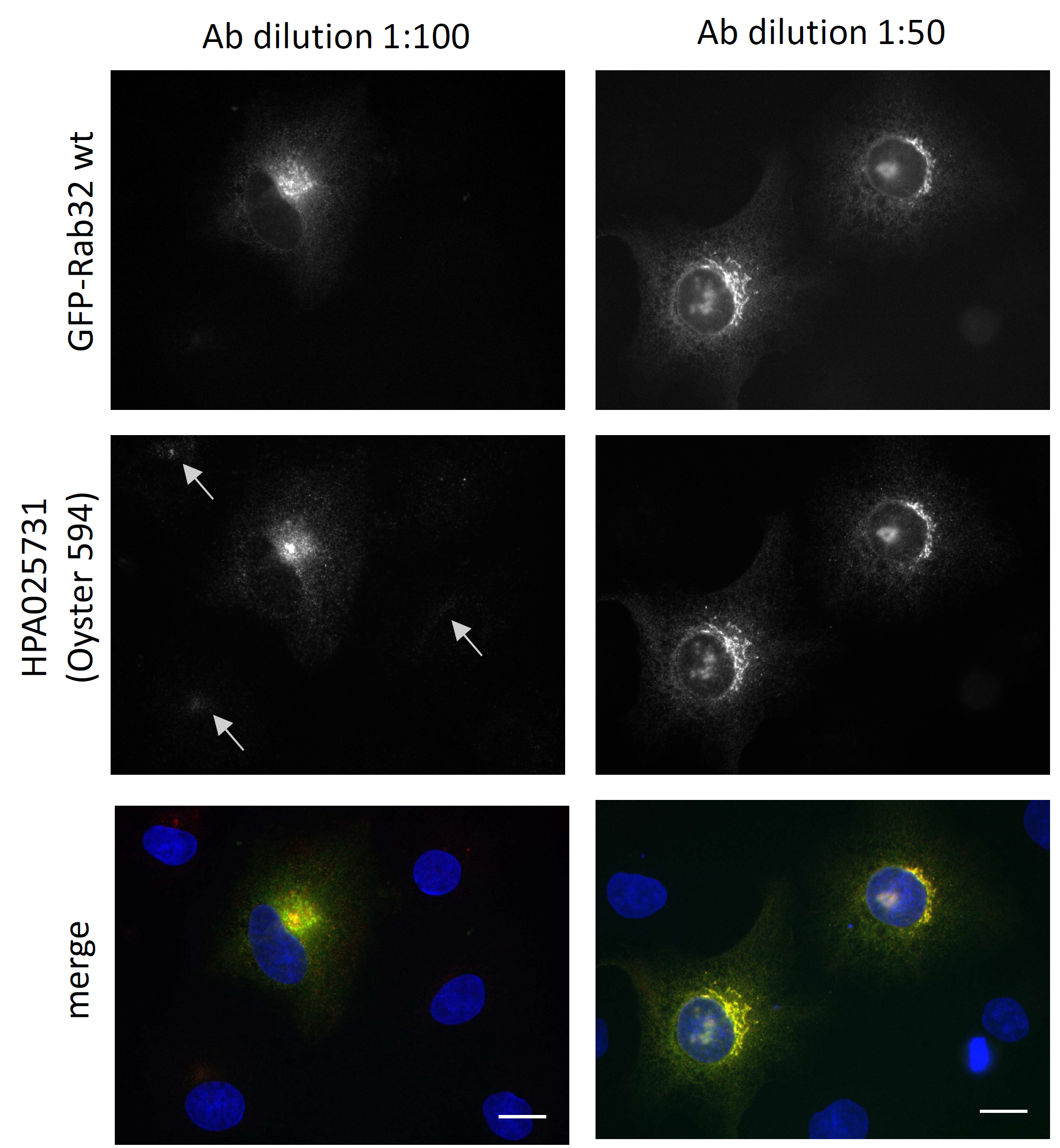

Supplement: S2 Fig — IHKE-1 cells stably expressing GFP-Rab32 wt were grown on glass cover slips for 24 hours followed by fixation and subsequently stained with an Rabbit anti Rab32 antibody (HPA025731). Despite being ‘stable’ some cells lost the expression of GFP-Rab32 wt–visible endogenous Rab32 was indicated by the arrow. Scale bar 10 μm. (TIF) [file pone.0208889.s003.tif]

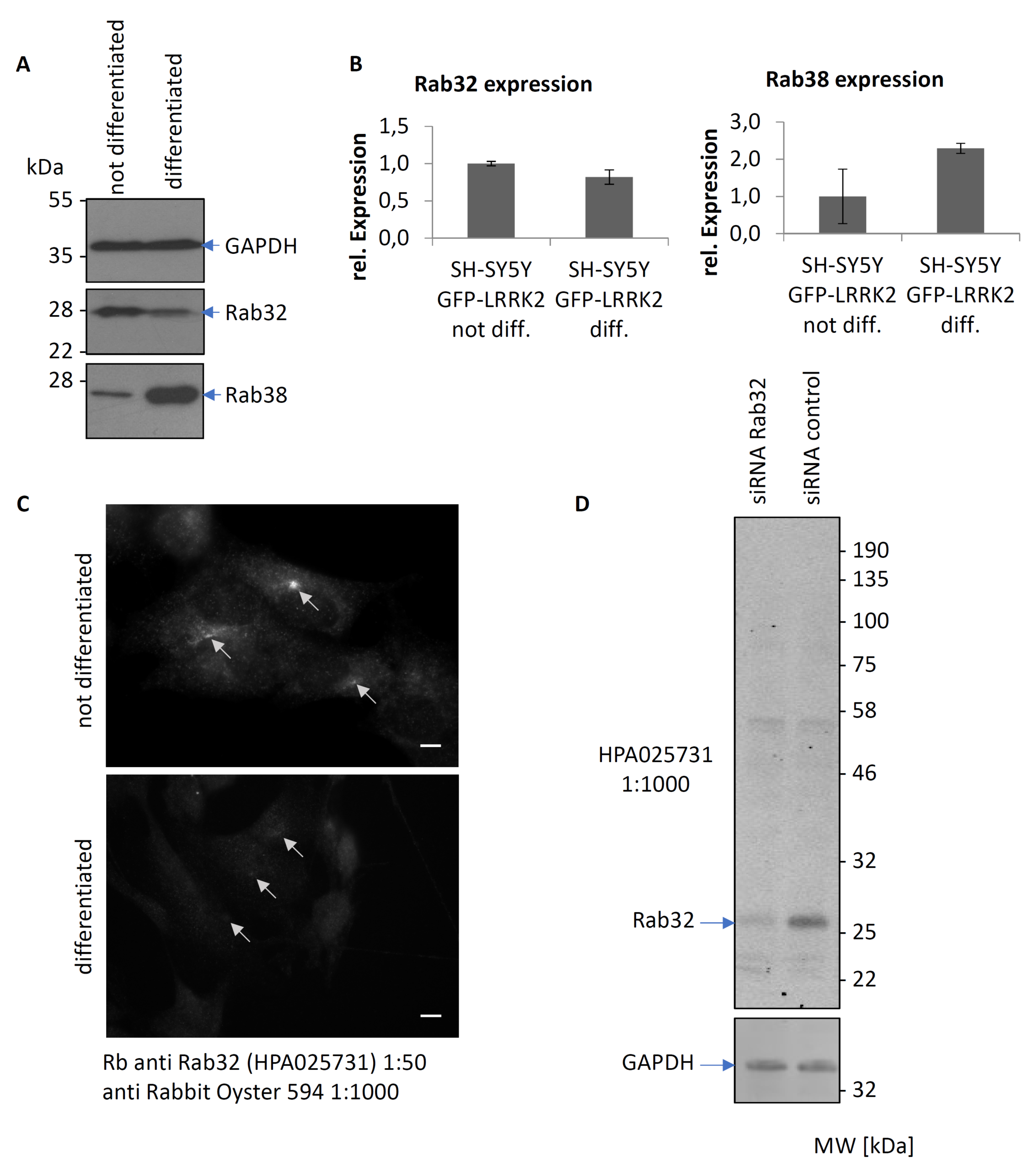

Supplement: S3 Fig — (A) SH-SY5Y cells were either grown normally or in the presence of 10 μM retinoic acid to induce neuronal differentiation. After lysis Western blots against Rab32 (Rabbit andt Rab32 SAB4200086), Rab38 and GAPDH as loading control were performed. (B) Quantification of the Western blots shown in (A); n = 3 independent experiments (C) Seconday immunofluorescence of SH-SY5Y cells stably expressing GFP-LRRK2 (not shown) either undiffentiated or differentiated by retinoic acid for 7 days. The images were taken at a 2 seconds exposure as 16 bit .tif files and the images were adjusted equally (black value was set to 900, white to 5700 of a total range of 0 to 65535). This allows a visual comparison of the signal strength. Scale bar = 10 μm (D) siRNA knockdown of Rab32. IHKE-1 cells were transfected with either control or siRNA against Rab32 for 3 days. Then lysates were prepared and and Western blots were done against GAPDH as loading control (lower panel) and Rab32 using the HPA025731 antibody (upper panel). (TIF) [file pone.0208889.s004.tif]

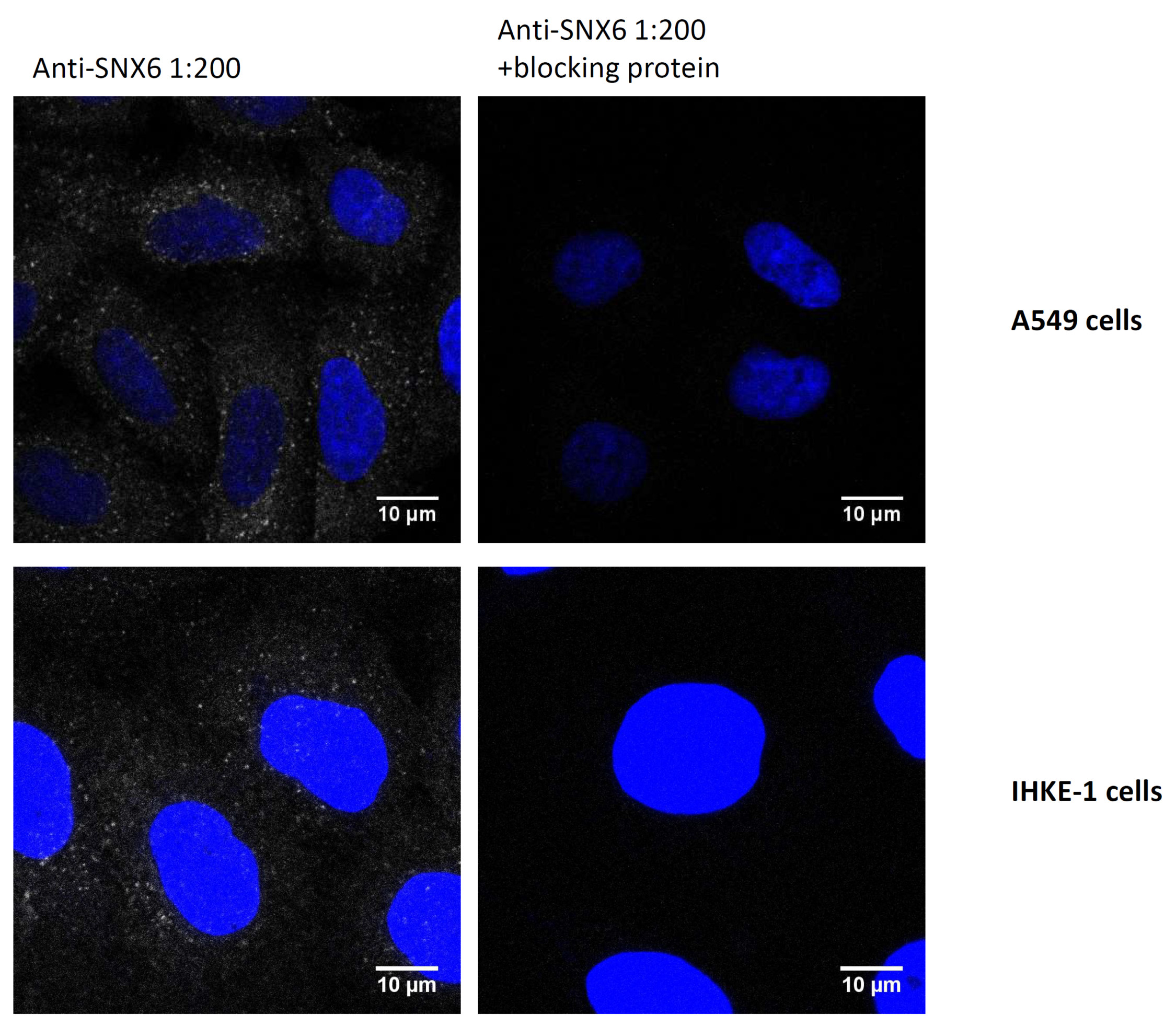

Supplement: S4 Fig — A549 (upper panel) or IHKE-1 cells were grown on glass cover slips before being fixed and stained for SNX6. In order to test the sepcificity of the antibody we added 0,35μg 6his-SNX61-193-construct to the primary antibody solution for 5 minutes. The control was without this protein. Both samples were incubated with the same amount of secondary antibody. Samples containing blocking protein and the respective controls were analyzed on a LSM5 microscope with equal settings for laser power, pinhole and detector gain. Scale bar = 10 μm; n = 3 independent experiments. (TIF) [file pone.0208889.s005.tif]

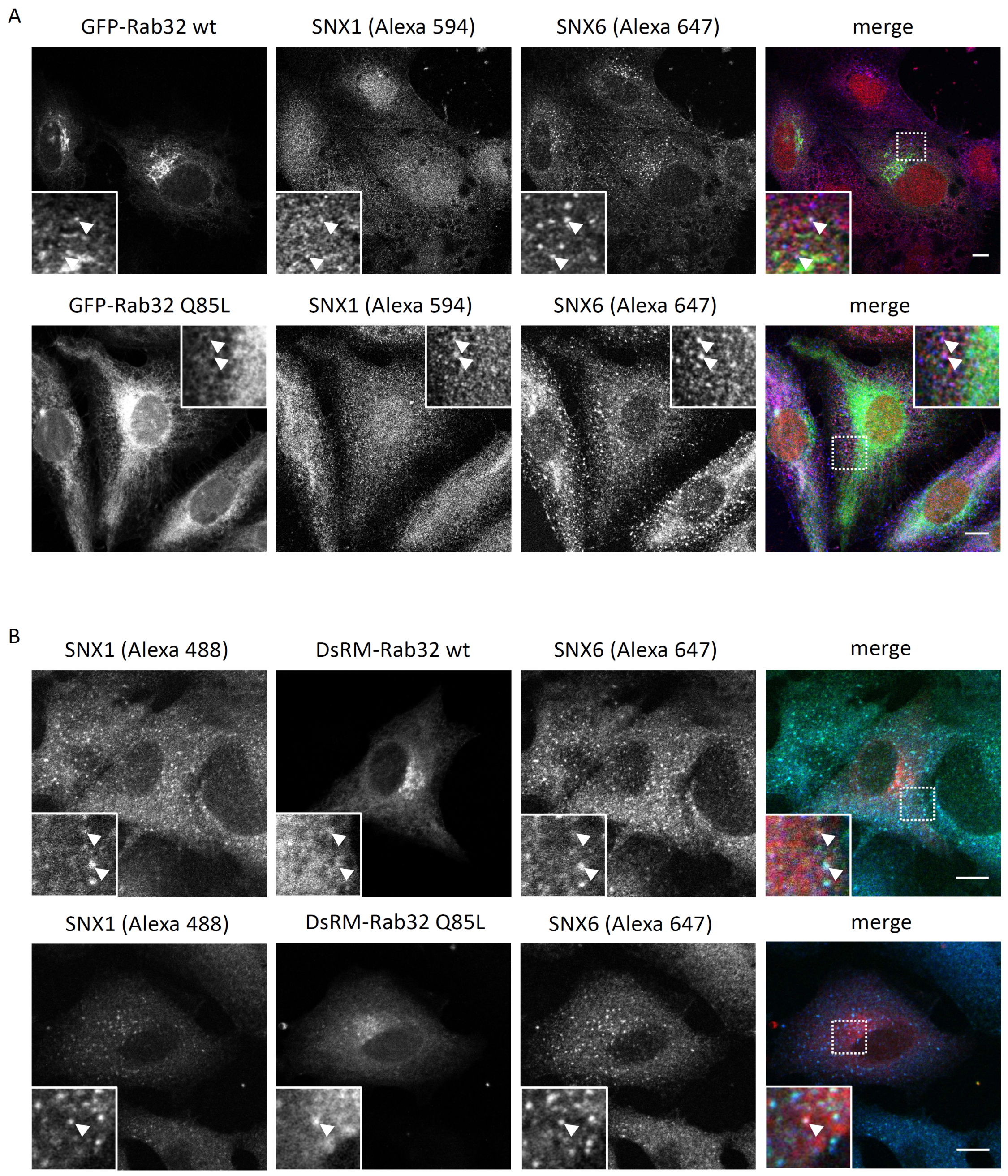

Supplement: S5 Fig — (A) IHKE 1 cells stably expressing either GFP-Rab32 wt (upper panel) or GFP-Rab32 Q85L (lower panel) were grown for 24 hours on glass cover slips. Then cells were fixed and stained for SNX1 and SNX6. Green channel: GFP; Red channel: Alexa 594 (SNX1); Blue channel: Alexa 647 (SNX6). Scale bar = 10μm (B) A549 cells were grown on glass cover slips for 24 hour followed by transfection with plasmids to express either DsRed-Monomer-Rab32 wt or DsRed-Monomer-Rab32 Q85L (depicted in red). After another 24 hours the cells were fixed and immunofluorescently labelled for SNX1 (Alexa488; green channel) and SNX6 (Alexa 647; blue channel). Scale bar = 10μm; (TIF) [file pone.0208889.s006.tif]

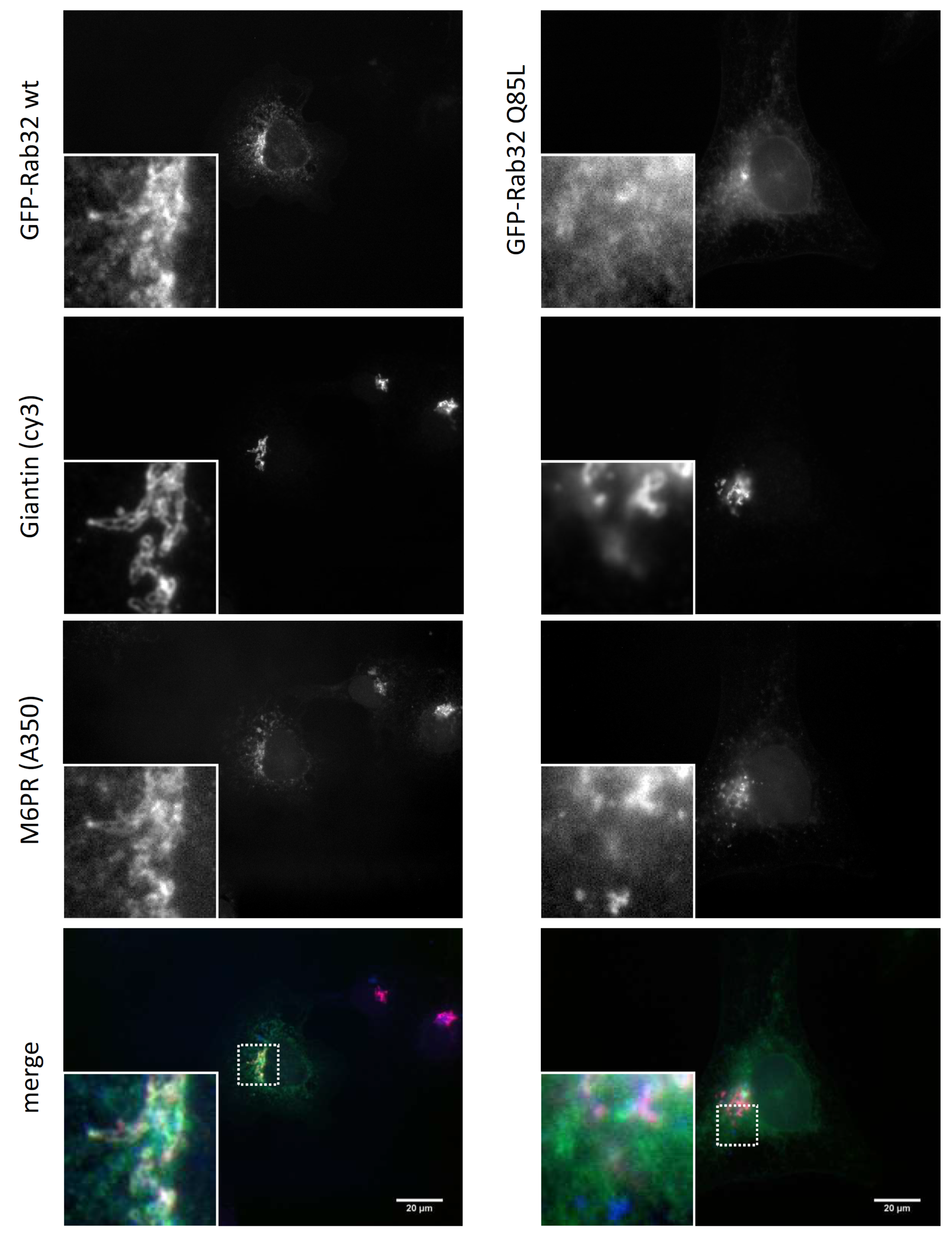

Supplement: S6 Fig — IHKE-1 cells stably expressing either GFP-Rab32WT or GFP-Rab32 Q85L wer grown on glass cover slips, fixed and immunofluorescently labelled against Giantin (red channel) and M6PR (blue channel). Scalebar = 20μm. (TIF) [file pone.0208889.s007.tif]

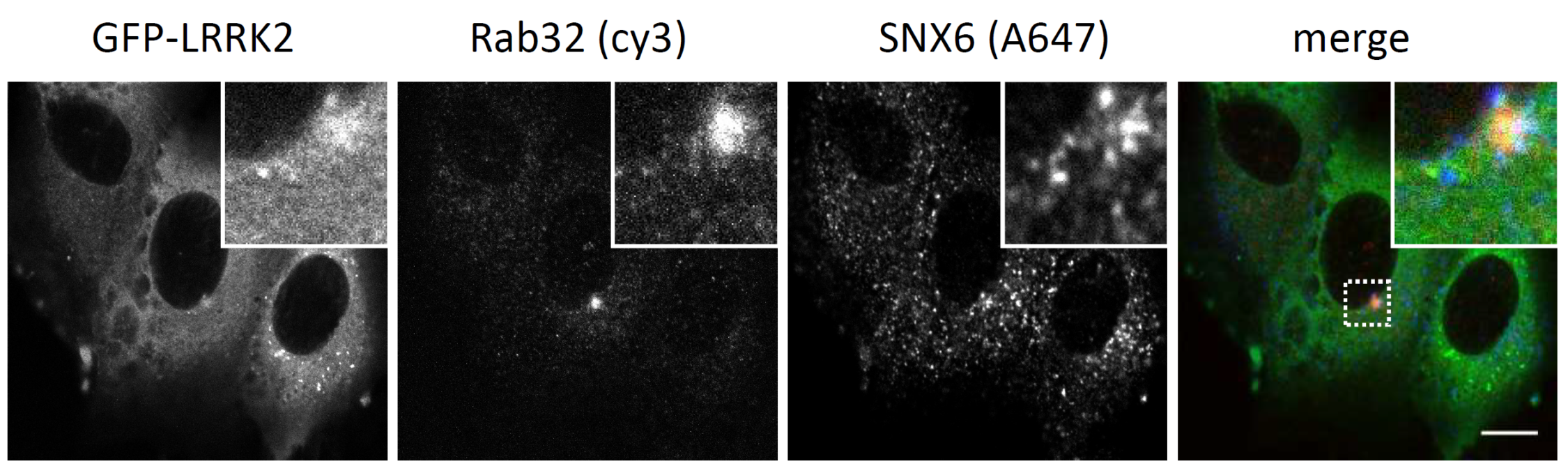

Supplement: S7 Fig — SH-SY-5Y stably expressing GFP-LRRK2 cells were cultured on glass cover slips for 48 hours. Then, cells were fixed and stained with antibodies against Rab32 and SNX6. Secondary antibodies were coupled to cy3 or Alexa 647. Cells were analyzed wit a Zeiss LSM5 microscope. n = 2 independent experiments. Colors in the merge image: GFP = green, cy3 = red, Alexa 647 = blue; Scale bar = 10μm. (TIF) [file pone.0208889.s008.tif]
