# Supplementary material for: Rab32 interacts with SNX6 and affects retromer-dependent Golgi trafficking
Source: PLoS One. 2019 Jan 14;14(1):e0208889. doi: 10.1371/journal.pone.0208889 (PMC6331118; doi:10.1371/journal.pone.0208889)
Supplement: S1 Table — In order to test whether constitutively active (Q85L) or inactive (T39N) mutants interact with SNX6, we co-transformed the yeast strain Gold with the indicated plasmids. Colony growth on QDO plates and blue color indicates that the proteins interact (+), n≥3 independent experiments. (DOCX) [file pone.0208889.s009.docx]

**Table S1. Nucleotide specificity of Rab32 binding SNX6**

| bait plasmid | prey plasmid | QDO + X-α-Gal + Aureobasidin A |
| --- | --- | --- |
| pAS 2-1-Rab32 Q85L | pGADT7-SNX6 | **+** |
| pAS 2-1-Rab32 wt |  | **+** |
| pAS 2-1-Rab32 T39N |  | **-** |
